# *In situ* synchrotron X-ray total scattering measurements and analysis of colloidal CsPb*X*_3_ nanocrystals during flow synthesis

**DOI:** 10.1107/S1600577523007300

**Published:** 2023-09-22

**Authors:** Matthew W. Greenberg, Cheng-Hung Lin, Shirish Chodankar, Sanjit K. Ghose

**Affiliations:** a Department of Chemistry and Biochemistry, Bard College, 30 Campus Road, Annandale-on-Hudson, NY 12504, USA; b National Synchrotron Light Source II, Brookhaven National Laboratory, Upton, NY 11973, USA; University of Malaga, Spain

**Keywords:** perovskites, nanocrystals, small-angle X-ray scattering, X-ray total scattering, nucleation, pair distribution function

## Abstract

*In situ* X-ray scattering measurements of CsPb*X*_3_ (*X* = Cl, Br, I) nanocrystal formation and halide exchange at NSLS-II beamlines were performed in an automated flow reactor. Total scattering measurements were performed at the 28-ID-2 (XPD) beamline and small-angle X-ray scattering at the 16-ID (LiX) beamline. Nanocrystal structural parameters of interest, including size, size distribution and atomic structure, were extracted from modeling the total scattering data. The results highlight the potential of these beamlines and the measurement protocols described in this study for studying dynamic processes of colloidal nanocrystal synthesis in solution with timescales on the order of seconds.

## Abstract 2

The formation of colloidal CsPb*X*_3_ perovskite nanocrystal in solution was interrogated with *in situ* X-ray total scattering/pair distribution function analysis in a flow cell reactor at the XPD 28-ID-2 beamline.

## 1. Introduction

Fully inorganic caesium lead halide perovskite nanocrystals have been explored as potential emissive materials in displays and LEDs due to their bright, tunable and narrow photoluminescence (Protesescu *et al.*, 2015 ▸; Akkerman *et al.*, 2015 ▸; Zhang & Yin, 2018 ▸). In particular, the defect tolerant band structure and ability to tune the bandgap by halide substitution make colloidal lead halide perovskite nanocrystals attractive as solution processible and tunable luminescent materials (Tao *et al.*, 2019 ▸; Brandt *et al.*, 2017 ▸). The synthesis of CsPb*X*_3_ (*X* = Cl, Br, I) nanocrystals from ionic metathesis of Cs^+^, Pb^2+^ and *X* ^−^ salts is known to proceed within seconds even at room temperature (Koolyk *et al.*, 2016 ▸; Li *et al.*, 2016 ▸; Nedelcu *et al.*, 2015 ▸), and is relatively facile compared with II–VI (García-Rodríguez *et al.*, 2013 ▸) and III–V (Tamang *et al.*, 2016 ▸) semiconductor nanocrystal synthesis. These rapid formation kinetics alongside structural lability of lead halide perovskites make mechanistic study of the nanoscale formation of these important luminescent materials a substantial experimental challenge (Kovalenko *et al.*, 2017 ▸).

Recently, flow synthesis and *in situ* photophysical characterization have been applied towards high-throughput studies of semiconductor nanocrystal formation (Lignos *et al.*, 2015 ▸; Abolhasani *et al.*, 2015 ▸; Epps *et al.*, 2017 ▸). Continuous-flow reactors afford fine control over reaction residence time and volumetric precursor ratios and can facilitate efficient screening of the large parameter space (*e.g.* reaction time, reagent and surfactant concentrations, temperature) of a nanocrystal synthesis (Abdel-Latif *et al.*, 2020 ▸). Several studies using flow reactors with in-line UV–vis absorbance/emission data collection have advanced mechanistic understanding and synthetic condition optimization for obtaining desired photophysical properties from lead halide perovskites (Maceiczyk *et al.*, 2017 ▸; Epps *et al.*, 2020 ▸; Abdel-Latif *et al.*, 2020 ▸).

Although electronic spectroscopy can provide quantitative information on lead halide perovskite nanocrystal concentration and size (Maes, Balcaen *et al.*, 2018 ▸), direct information on ensemble atomic structure during the nanocrystal formation process is essential for mechanism-guided synthesis. Time-resolved *in situ* diffraction and scattering measurements of nanocrystal formation capable of providing this atomic structural information have been made possible by the brilliance of X-ray light available at third-generation synchrotron radiation user facilities (Wu *et al.*, 2019 ▸). Total X-ray scattering has been especially important for structural studies of lead halide perovskite nanocrystals in order to distinguish closely related crystalline phases (cubic, tetragonal and ortho­rhombic), characterize the substantially labile surface structure, and reveal nanoscale twinning effects (Cottingham & Brutchey, 2016 ▸; Bertolotti *et al.*, 2017 ▸, 2019 ▸).

X-ray total scattering and real-space pair distribution function (PDF) analysis is a key approach for studying complex nanostructure as it is capable of measuring both average and local atomic structure (Banerjee *et al.*, 2018 ▸; Christiansen *et al.*, 2020 ▸). By Fourier transforming Bragg diffraction and diffuse scattering along a wide range of momentum transfer *Q*, a pair correlation function *G*(*r*) is produced which measures the deviation from the average number density at a pair distance *r* (Farrow & Billinge, 2009 ▸). This method can extract structural information from dilute nanocrystals in solution from a difference PDF method (Terban *et al.*, 2015 ▸). *In situ* PDF analysis has been used for measuring atomic-scale structural information during the nanocrystal formation process in many studies of structural evolution in the solvothermal synthesis of metal oxides (Saha *et al.*, 2014 ▸; Jensen *et al.*, 2012 ▸; Dippel *et al.*, 2016 ▸). Atomistic and virtual crystal modeling of real-space *G*(*r*) can be used to extract quantitative atomic structural data during prenucleation and nucleation and growth stages of synthesis (Bøjesen & Iversen, 2016 ▸; Dippel *et al.*, 2016 ▸; Bertolotti *et al.*, 2018 ▸; Campos *et al.*, 2022 ▸). Alongside the atomic local structure from the real-space PDF analysis, information on the nanocrystal shape, average size and size dispersity can be extracted from the Debye scattering equation (DSE) fitting of total scattering in reciprocal space (Cervellino *et al.*, 2015 ▸). This provides an alternative to separate transmission electron microscope (TEM) or small-angle X-ray scattering (SAXS) data collection as is most often used for establishing nanocrystal shape and size distribution information (Pyrz & Buttrey, 2008 ▸; Maes, Castro *et al.*, 2018 ▸). Therefore, as a complementary analysis to real-space modeling of *G*(*r*), DSE fitting of *I*(*Q*) data with high-reciprocal-space resolution can provide nanostructure information.

**Figure 1:**
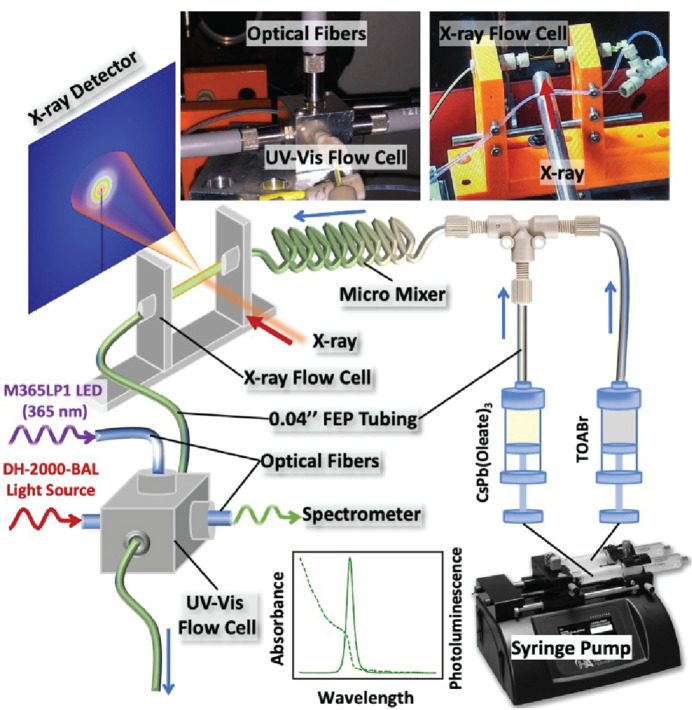
Flow reactor setup for in-line total X-ray scattering and UV–Vis measurements at XPD 28-ID-2 beamline.

Here, we report total scattering measurements for colloidal lead halide perovskites during synthesis in a custom-designed continuous-flow reactor (Fig. 1 ▸). The control of residence times and precursor ratios by adjusting the relative and absolute precursor flow rates allows us to explore both reaction dynamics and the influence of different precursor concentrations on final nanocrystal structure. Stopped-flow and continuous-flow reactors have been applied for X-ray scattering measurements of nanocrystal formation on millisecond and second timescales in the study of gold nanocrystal formation using SAXS (Abécassis *et al.*, 2007 ▸; Polte *et al.*, 2010 ▸). Benchmarking similar reactors at synchrotron facilities for measuring X-ray total scattering of nanoparticles in solution during synthesis will help reveal structural evolution at the atomic and nano size scales during these processes.

## 2. Experimental

*In situ* synthesis of CsPb*X*_3_ was performed in a modified flow synthesis setup (Fig. 1 ▸) adapted from Epps *et al.* (2017 ▸). Stock solutions were prepared following a room-temperature synthesis of CsPbBr_3_ from reacting *in situ* generated lead(II) oleate and caesium oleate with tetraoctyl­ammonium bromide (Wei *et al.*, 2016 ▸). Stock solution concentrations were [Cs^+^] = [Pb^2+^] = [Br^−^] = 30 m*M* in 1:5 oleic acid:toluene for collecting total X-ray scattering data. The precursor solutions were prepared at 6 m*M* for the SAXS measurements to limit nanocrystal aggregation. Zinc halide (Cl, I) solutions were formed from dissolving the zinc halide in 1:19 oleyl­amine:toluene at a concentration of 60 m*M* [Zn^2+^]. Steel syringes containing Cs^+^/Pb^2+^ and Br^−^ stock solutions were loaded into Harvard Apparatus PHD Ultra syringe pumps and injected into 0.040 inch inner-diameter fluorinated ethyl­ene propyl­ene (FEP) tubing and then mixed by combining in poly(ether ether ketone) (PEEK) T-joints. For the halide exchange experiments, the zinc halide solution described above was combined with flow-synthesized CsPbBr_3_ by a second T-joint using an independent Harvard Apparatus DDS dual syringe pump. Braided FEP tubing micromixers were used to assure mixing of the precursor streams. The final mixed solution was fed to a flow cell, placed in the X-ray beam path for scattering data collection. In-line photoluminescence was recorded by excitation with a ThorLabs 365 nm LED (M365LP1) through fiber optic cables and an Ocean Insight QE Pro spectrometer with spectral range from 200 nm to 990 nm. UV–Vis and photoluminescence data were collected with a 75 ms integration time per scan. The UV–Vis flow cell was a custom-designed aluminium flow cell with SMA fiber optic connectors made to simultaneously monitor the absorption and emission spectrum during flow. The reaction residence time could be controlled by tuning two parameters, one being the flow rate and the other the reactor path length from mixer to the probe spot (X-ray and optical) in the flow cell. For this synthesis of CsPb*X*_3_ nanocrystals we have demonstrated the consistent control of residence time using different flow rates as well as mixer lengths.

X-ray total scattering measurements were performed at Brookhaven National Laboratory using the 28-ID-2 (XPD) high-energy X-ray powder diffraction beamline at the National Synchrotron Light Source II (NSLS-II). The fully automated flow reactor setup is integrated into the controls system (EPICS), and data collection and inline data reduction and visualization is carried out using the *Bluesky* suite Python programming language-based protocols (Koerner *et al.*, 2020 ▸; Allan *et al.*, 2019 ▸; Bluesky Project, https://blueskyproject.io/). X-ray total scattering data were collected in rapid acquisition mode using a 2D PerkinElmer detector (2048 × 2048 pixels, 200 µm × 200 µm per pixel) and a sample-to-detector distance of 254 mm. The incident energy of the X-rays was 67.13 keV (λ = 0.1847 Å). *In situ* synthesized samples were measured in 1.5 mm Kapton polyimide tubes in the X-ray flow cell shown in Fig. 1 ▸. An Ni standard was used as a calibrant. Azimuthal integration from raw 2D detector intensities to the 1D *I*(*Q*) was performed using *pyFAI* (Ashiotis *et al.*, 2015 ▸). Background scattering subtraction, and normalization and corrections to generate the total scattering structure function *F*(*Q*) and its Fourier transform to the PDF *G*(*r*) were performed using *PDFgetX3* in *xPDFsuite* (Yang *et al.*, 2014 ▸; Juhás *et al.*, 2013 ▸). *Q*_min_ was determined by the beamstop at 0.45 Å^−1^ and *Q*_max_ to reduce statistical noise at 20 Å^−1^. Small-box PDF modeling was performed using *PDFgui* (Farrow *et al.*, 2007 ▸) and structures from the materials project library (mp-600089, mp-567629) (Jain *et al.*, 2013 ▸). DSE method fitting of the data in reciprocal space was performed using *DEBUSSY* (Cervellino *et al.*, 2015 ▸). The instrumental resolution function for the *DEBUSSY* software was corrected by convoluting the calculated DSE with a pseudo-Voigt function (Dengo *et al.*, 2022 ▸) with parameters used from nickel metal powder standard from NIST using *TOPAS v7.17* (Coelho, 2018 ▸). SAXS was performed at NSLS-II on the Life Sciences X-ray Scattering (LiX) beamline 16-ID. Data were collected at a wavelength of 0.819 Å using the detectors Pilatus 1M in air and Pilatus 900K in vacuum with a pixel size of 172 µm; both detectors record data simultaneously to cover a wide contiguous *q* range. This configuration yielded an accessible scattering range of 0.006 < *q* < 3.0 Å^−1^, where *q* is the momentum transfer, defined as *q* = 4πsin(θ)/λ (λ is the wavelength and 2θ is the scattering angle). Calibration of the detectors was carried out using silver behenate, which has a lamellar structure with ∼5.8 nm spacing.

## 3. Results and discussion

**Figure 2:**
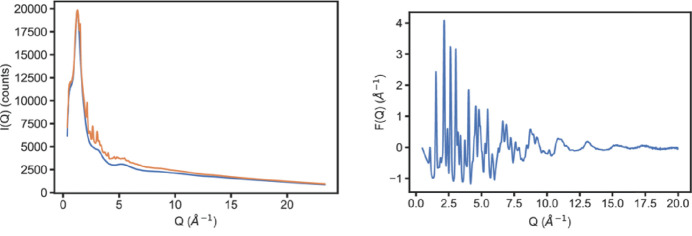
(Left) Azimuthally integrated intensity of flow-synthesized CsPbBr_3_ nanocrystals in toluene/oleic acid (orange) with separately measured solvent scattering (blue). Each *I*(*Q*) curve is generated from a 10 min data acquisition. (Right) Normalized reduced total scattering structure function *F*(*Q*) of nanocrystals extracted from the figure on the left.

Caesium lead bromide perovskite nanocrystals were synthesized in flow by a previously reported room-temperature route from caesium lead oleate and tetraoctyl­ammonium bromide precursors adapted for flow chemistry (Epps *et al.*, 2017 ▸). Combining 30 m*M* Cs+/Pb^2+^/Br^−^ solutions in the flow reactor described above resulted in solutions of nanocrystals with clearly visible Bragg reflections in reciprocal space as is shown in Fig. 2 ▸ (left). The datasets shown in Fig. 2 ▸ are of a completed nanocrystal formation reaction collected with a 60 cm total reactor path length and 100 µl min^−1^ flow rate for both precursors. Collection of data at a given reaction path distance under continuous flow may be performed to ensure good signal to noise in *F*(*Q*) of the nanocrystals extracted from subtracting the background solvent scattering as is shown in Fig. 2 ▸ (right). Under these conditions the *G*(*r*) generated from this *F*(*Q*) is fit excellently to a CsPbBr_3_ nanocrystal model and can also be fit in reciprocal space, as is shown later in Figs. 4 and 5.

**Figure 3:**
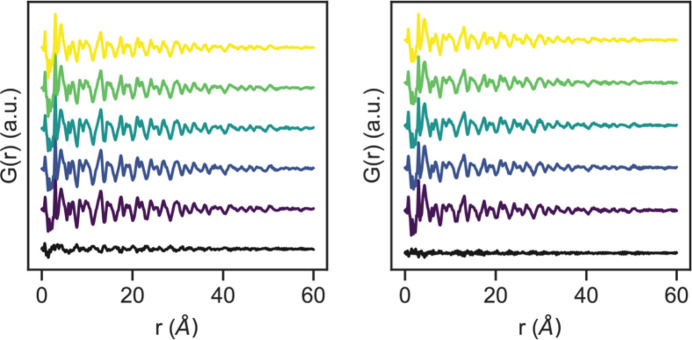
*G*(*r*) of flow-synthesized CsPbBr_3_ nanocrystals from consecutive 1 min scans at constant flow rates [250 (left) and 750 µl min^−1^ (right)]. The black difference curve at the bottom of each plot is the difference between the final and first scan in the figure.

As mentioned previously, the rapid kinetics associated with the solution formation of CsPbBr_3_ nanocrystals has made studying the structural evolution during these processes an experimental challenge (Koolyk *et al.*, 2016 ▸). Flow chemistry alongside in-line UV–Vis data sampling has previously been used to map solution formation processes of perovskite materials, as residence time can be controlled by either adjusting flow rates or measuring at different positions along the reactor (Lignos *et al.*, 2020 ▸; Abdel-Latif *et al.*, 2020 ▸). Using our experimental reactor, by varying the flow rates and continuously collecting diffraction images we can measure different time points during the formation of the CsPbBr_3_ nanocrystals as is shown in Fig. 3 ▸. The datasets shown in this figure consist of *G*(*r*) traces generated from 1 min scans under continuous flow at two different flow rates, 250 and 750 µl min^−1^, with a 15 cm path length reactor. Under these conditions, the higher flow rate and path lengths can be used to isolate reaction timepoints following nucleation during the nanocrystal growth process which is discussed in detail later. For each flow rate, *G*(*r*) from consecutive scans shows a high degree of similarity as illustrated by the black difference plot between the first and final scan in the series. Therefore, we see here that the flow reactor allows for continuous X-ray scattering data to be collected at a given residence time.

**Figure 4:**
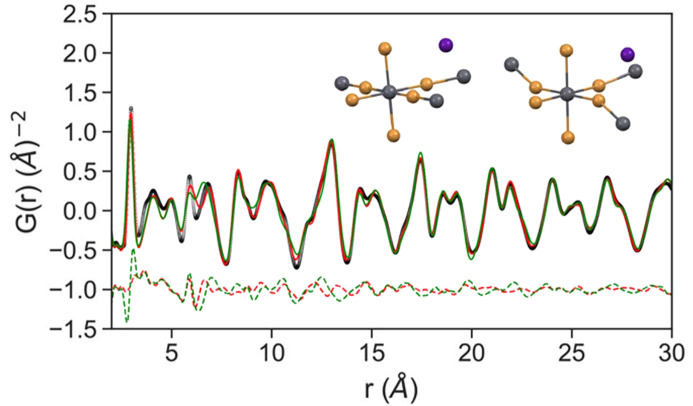
CsPbBr_3_ nanocrystals (black circles) alongside orthorhombic fit (*Pnma*, red line) and cubic fit ( 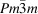 , green line). *G*_diff_ = (*G*_obs_ − *G*_calc_) for each fit is plotted in dashed lines offset below the data. The octahedral coordination environment of Pb(II) ions for *Pnma* and 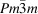 structures are shown in the insets from left to right (Pb = gray, Br = yellow, Cs = purple).

Real-space PDFs of flow-synthesized nanocrystals are fit using the ‘real space Rietveld’ type structural refinement in *PDFgui* (Farrow *et al.*, 2007 ▸). While bulk CsPbBr_3_ is known to adopt an orthorhombic crystal structure, nanocrystals of CsPbBr_3_ have been assigned as either cubic or orthorhombic based on assigning broad nanocrystal diffraction from laboratory X-ray diffraction (Protesescu *et al.*, 2015 ▸; Cottingham & Brutchey, 2016 ▸). The difference between the two closely related structures can be visualized as distortions of the Pb coordination environment from octahedral symmetry in the lower symmetry crystal structure as is shown in Fig. 4 ▸. Using the real-space difference PDFs of the nanocrystal structure we examined the agreement of the *Pnma* model, which is substantially better than the 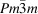 model, and relevant structural parameters and goodness of fit *rw* are detailed in Table 1 ▸. The orthorhombic model appears to be a clearly superior model for the local structure of the nanocrystals as compared with the cubic, both by comparison of *rw* values and the slightly unphysically large atomic displacement parameter (ADP) values refined for the 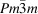 model. The *rw* fit value of 0.174 for the preferred ortho­rhombic model is within the range of good fits for small-box PDF modeling of nanocrystals (Banerjee *et al.*, 2018 ▸). More detailed synchrotron diffraction and real-space PDF studies of CsPbBr_3_ nanocrystals, like our results, have consistently assigned the structure of the nanocrystals as locally ortho­rhombic (Cottingham & Brutchey, 2016 ▸; Bertolotti *et al.*, 2017 ▸).

**Figure 5:**
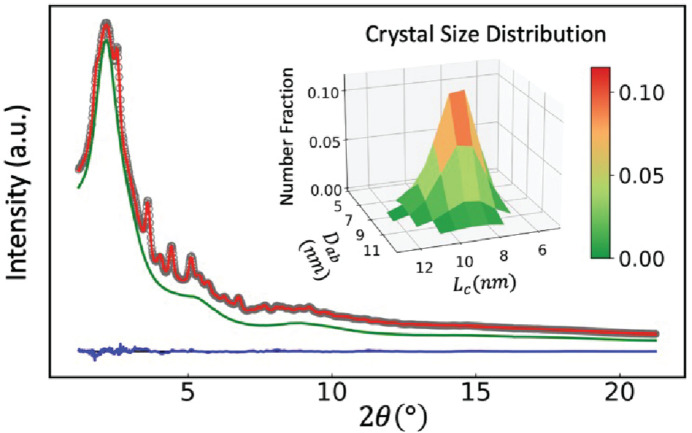
Total X-ray scattering data (gray circles) acquired at the XPD 28-ID-2 beamline and DSE best fit (red line) of *in situ* synthesized CsPbBr_3_ colloidal nanocrystals in toluene solvent (green line). The difference between DSE simulation and experimental data is shown by the blue line. The inset describes the 3D representation of the bivariate log-normal crystal size distribution function.

This assignment is further corroborated by fitting the total scattering data in reciprocal space using the DSE method as implemented in *DEBUSSY*, as shown in Fig. 5 ▸. The DSE model used to fit the data in Fig. 5 ▸ corresponds to an ortho­rhombic structure with a parallelepiped shape. The parallelepiped defines two independent spatial directions as fitting parameters, one along the *ab* plane and one along *c*. The simulated crystal size distribution is then summarized as an inset in Fig. 5 ▸. The DSE fitting results of the *Pnma* model and the 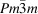 model are also summarized in Table 1 ▸. The reciprocal-space fitting of particle shape and dispersity by the DSE method here is consistent with the morphology and size dispersity reported previously for this room-temperature synthesis by TEM measurements (Wei *et al.*, 2016 ▸). In both real- and reciprocal-space fitting of the total scattering data the orthorhombic structure provided a better fit to experimental data than the higher-symmetry cubic structure for nanocrystals synthesized in our room-temperature flow synthesis conditions. This is consistent with the previously mentioned synchrotron X-ray studies of CsPbBr_3_ nanocrystals synthesized from high-temperature hot injection syntheses (Cottingham & Brutchey, 2016 ▸; Bertolotti *et al.*, 2017 ▸), which suggests that this preference for the lower symmetry ortho­rhombic crystal phase is likely general for nanocrystals of CsPbBr_3_.

**Figure 6:**
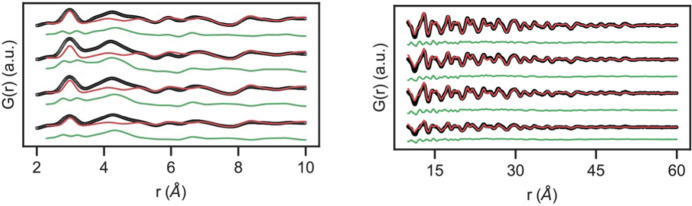
*G*(*r*) data (black) recorded at different precursor flow rates (1000, 750, 500 and 250 µl min^−1^ bottom to top with a 15 cm reactor length), alongside the *Pnma* nanocrystal fit (red) and difference (green). The low-*r* fit is shown on the left and high-*r* on the right.

**Figure 7:**
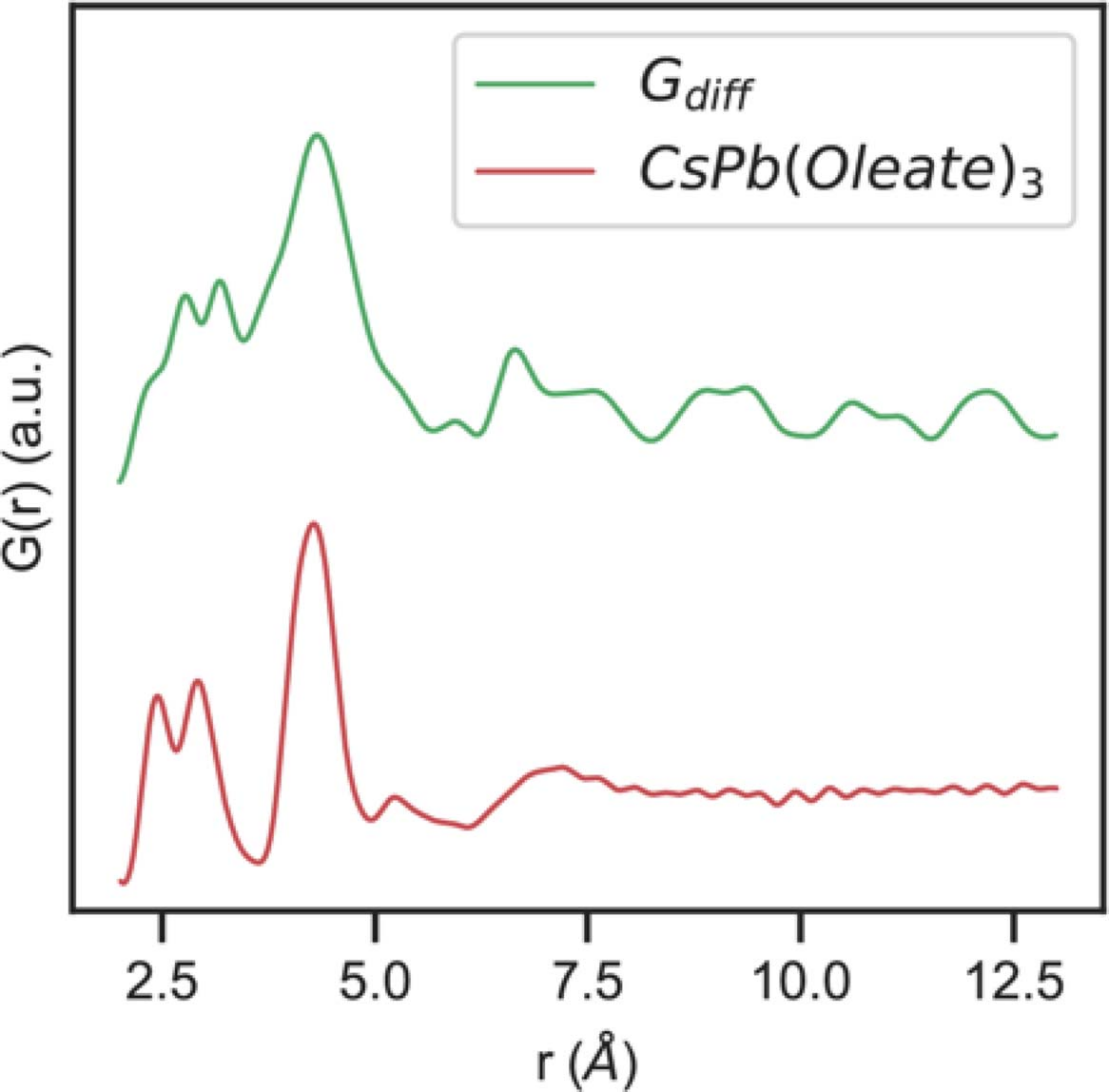
*G*(*r*) data for the CsPb(oleate)_3_ precursor (bottom red trace) and *G*_diff_ for the CsPbBr_3_ virtual crystal model and data for the 1000 µl min^−1^ reaction conditions shown in Fig. 6 ▸.

The crystalline fraction of CsPbBr_3_ during intermediate stages of nanocrystal formation can also be modeled using the virtual crystal model approach in *PDFgui*. These data are shown in Fig. 6 ▸ using 1000, 750, 500 and 250 µl min^−1^ flow rates. The growth of the nanocrystal can be seen by the appearance of increasingly intense peaks at higher pair distances, consistent with what has been reported in previous PDF studies on nanocrystal solution growth (Campos *et al.*, 2022 ▸). Consistent with other studies, the virtual crystal model accounts only partially for the observed *G*(*r*) which contains signal from molecular precursors, solutes and nucleated crystals that all coexist during the initial stages of nanocrystal formation. We fit low-*r* (0.23 up to 1 nm) and high-*r* (1–6 nm) regions separately, as the former region is most likely to contain real-space correlations from poorly ordered molecular precursors and solutes. The difference PDFs (green curves in Fig. 6 ▸) between the virtual crystal model and the data sets primarily show deviations below 1 nm in the low-*r* region (Table 2 ▸). This is presumably from the disordered Cs and Pb oleate molecular precursors and solute of CsPbBr_3_ that are present as the nanocrystals are formed. A separate X-ray PDF measurement of the 30 m*M* molecular Cs and Pb oleate precursor solution shows a strong correlation that aligns with the major peak that the virtual crystal model fails to capture slightly above 4 Å in the early time experimental *G*(*r*) as shown in Fig. 7 ▸. Prior X-ray PDF studies of related fatty acid lead carboxyl­ates in the literature assign the most prominent feature in these compounds that is seen in *G*(*r*) around this real-space distance as a Pb–Pb separation (Campos *et al.*, 2022 ▸; Martínez-Casado *et al.*, 2017 ▸). As expected, monotonic growth of the nanocrystals is seen in the increasing refined crystallite size refined in the high-*r* fitting as the flow rate is decreased at a given reactor length (Table 2 ▸). Consistent with previous reports on CsPbBr_3_ nanocrystals, our measurements are consistent with a virtually immediate nucleation of crystalline CsPbBr_3_ following mixing of the precursor solutions followed by a rapid nanocrystal growth process that we can observe different stages of by varying the total reaction residence time at a given reactor length by varying the precursor solution flow rates.

**Figure 8:**
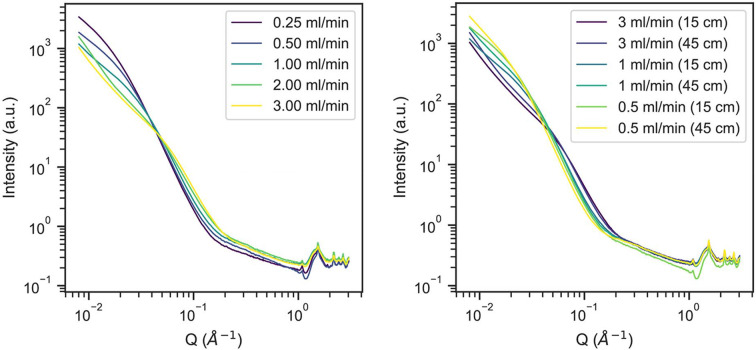
Small-angle X-ray scattering of CsPbBr_3_ nanocrystals formed in flow with varied residence times achieved by varying the flow rate of the precursors (left) at a constant reactor path length (15 cm) and by varying the reactor path length (right) at several different flow rates.

Consistent with the known fast nucleation kinetics of CsPbBr_3_, we see that even at room temperature we cannot observe an initial prenucleation stage as nanocrystalline orthorhombic CsPbBbr_3_ is observed in *G*(*r*) even at our fastest pump rate (an approximate residence time of 4 s in the reactor). We sought to further benchmark our reactor apparatus in capturing the formation process of CsPbBr_3_ by performing SAXS measurements. SAXS experiments were run at slightly lower precursor concentrations (6 m*M*) in order to avoid excessive particle aggregation, and further confirm the rapid formation of final nanocrystals (Fig. 8 ▸) at room temperature. Representative datasets shown in Fig. 8 ▸ demonstrate the effect of altering the flow rate at a given reactor length (Fig. 8 ▸, left), as well as the reactor length for several different flow rates (Fig. 8 ▸, right). The increase in intensity as *Q* approaches zero and shift of the position of the decay of intensity to lower *Q* are both a consequence of the increasing size of nanocrystals and concentration of CsPbBr_3_ in nanocrystals as the reaction residence time increases. The uptick at low *Q* in all of the datasets in Fig. 8 ▸ represents rapid interparticle aggregation under all recorded conditions shown below. Finally, several sharp Bragg peaks consistent with CsPbBr_3_ seen in the high-*Q* region of the data between *Q* = 1 and 3 Å^−1^ corroborate the identity of the nanocrystalline material.

**Figure 9:**
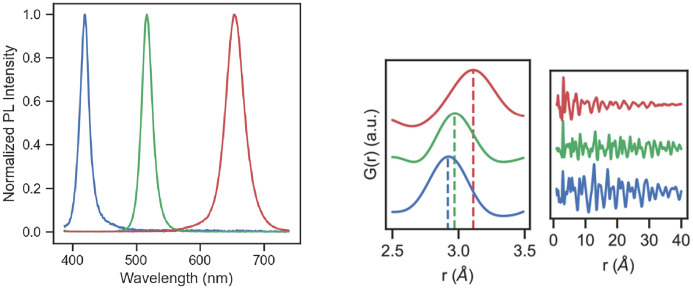
(Left) In-line photoluminescence spectroscopy of the Cl (blue) and I (red) exchanged CsPbBr_3_ (green) nanocrystals, along with local and long-range *G*(*r*) (center and right, respectively). Local first nearest neighbor and full *G*(*r*) of CsPb*X*_3_ nanocrystals formed from halide exchange. The dotted lines show the shift in first-nearest-neighbor correlation in going from Cl exchanged nanocrystals (blue), parent Br (green) and finally I exchanged nanocrystals (red).

Finally, we sought to observe the effects of halide substitution into CsPbBr_3_ nanocrystals synthesized in flow that could be observed by mixing a third concentrated precursor stream of ZnI_2_/ZnCl_2_ dissolved in oleyl­amine and toluene (Abdel-Latif *et al.*, 2019 ▸). The halide exchange reaction is confirmed by the shift of the perovskite photoluminescence (Fig. 9 ▸, left) that is also clearly visible by eye in the reactor. As expected from the relative atomic radii of the halogens, addition of *I* ^−^ to the CsPbBr_3_ lattice shifts the nearest-neighbor correlation to higher *r* and likewise to lower *r* for the addition of Cl^−^ (Fig. 9 ▸, center). Interestingly, the structural coherence of the nanocrystals is more substantially affected by the addition of iodide than chloride at the same relative concentration of iodide as is seen by the diminished intensity of correlations at higher *r* (Fig. 9 ▸, right). This may be due to different amounts of partial phase segregation during the initial stage of halide substitution into CsPbBr_3_ with either Cl^−^ or I^−^ (Gratia *et al.*, 2016 ▸; Zhang *et al.*, 2019 ▸). A more detailed follow-up study on structural changes during the process of halide substitution into CsPbBr_3_ will be published elsewhere to examine different possible explanations for the loss of structural coherence following iodide exchange seen here.

## 4. Conclusions

We have used X-ray total scattering at the NSLS-II XPD 28-ID-2 beamline to measure the atomic local structure by the real-space PDF method of dilute CsPbBr_3_ nanocrystals synthesized in flow. We further demonstrated that these measurements could be carried out on the nucleation and growth processes of these nanocrystals, as well as during the process of halide exchange reactions. Analysis of reciprocal-space total scattering data using the Debye scattering equation can be used to acquire complimentary data on morphology and the size distribution function. These results demonstrate that time-resolved X-ray total scattering measurements of dilute nanocrystals in solution during nucleation and growth processes may be performed to generate PDFs with high real-space resolution by controlling the residence time at the measurement point to allow for extended data collection. This is even the case when the underlying kinetics of the crystal growth process are shorter than the measurement time needed to acquire good signal to noise in reciprocal space which is essential for application of the real-space PDF method. The flow reactor principle explored in this work could therefore be of general interest for applying synchrotron X-ray total scattering measurements of atomic local structure on relatively fast nanocrystal nucleation and growth processes on the seconds to minutes timescales.

## Figures and Tables

**Table 1:** Refined parameters from *DEBUSSY* and *PDFgui* fitting Refined full-*r* (2–60 Å) fitting parameters and goodness of fit *rw* from *PDFgui* fits. δ_2_ is a 1/*r* ^2^ peak sharpening factor accounting for correlated atomic motion (Jeong *et al.*, 1999 ▸) used in the *PDFgui* fits. Averaged diameter and GoF are from the reciprocal-space *DEBUSSY* fitting. The same refined ADPs and lattice parameters from the full-*r* *PDFgui* fit are used in the *DEBUSSY* fitting, and the crystal size distribution as described above in Fig. 5 ▸ is refined.

Space group	*Pnma*	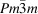
*a* (Å)	8.262	5.844
*b* (Å)	11.779	–
*c* (Å)	8.208	–
δ_2_	6.9	7.6
Cs *U* ^iso^ (Å^2^)	0.080	0.104
Pb *U* ^iso^ (Å^2^)	0.027	0.028
Br_1_ *U* ^iso^ (Å^2^)	0.069	0.164
Br_2_ *U* ^iso^ (Å^2^)	0.039	–
*rw*	0.174	0.275
Average diameter (nm)	10.50 ± 1.01	8.29 ± 3.73
GoF	0.376	0.601

**Table 2:** Selected fitting parameters from the PDF fits shown in Fig. 6 ▸

Flow rate (µl min^−1^)	*rw* (high-*r*)	*rw* (low-*r*)	*d* (Å)
1000	0.35	0.84	76.8
750	0.29	0.80	87.2
500	0.34	0.88	96.4
250	0.20	0.66	113.6
